# Interoceptive awareness in a Norwegian population: psychometric properties of the Multidimensional Assessment of Interoceptive Awareness (MAIA) 2

**DOI:** 10.1186/s12888-023-04946-y

**Published:** 2023-07-10

**Authors:** Charlotte Fiskum, Trine Tetlie Eik-Nes, Hamed Abdollahpour Ranjbar, Jannicke Andersen, Mojtaba Habibi Asgarabad

**Affiliations:** 1 Department of Psychology, Norwegian University of Science and Technology, Trondheim, Norway; 2 Department of Neuromedicine and Movement Science, Norwegian University of Science and Technology, Trondheim, Norway; 3 Stjørdal Community Mental Health Centre, Levanger Hospital, Nord-Trøndelag Hospital Trust, Levanger, Norway; 4 Department of Psychology, Koç University College of Social Sciences and Humanities, Istanbul, Turkey; 5 Department of Psychology, University of Oslo, Oslo, Norway; 6 Health Promotion Research Center, Iran University of Medical Sciences, Tehran, Iran; 7 Department of Health Psychology, School of Behavioral Sciences and Mental Health (Tehran Institute of Psychiatry), Iran University of Medical Sciences, Tehran, Iran; 8 Center of Excellence in Cognitive Neuropsychology, Institute for Cognitive and Brain Sciences, Shahid Beheshti University, Tehran, Iran; 9 Positive Youth Development Lab, Human Development & Family Sciences, Texas Tech University, Texas, USA

**Keywords:** Interoception, MAIA-2, Interoceptive awareness, Interoception and health, Body trust, Interoception and gender, Norwegian

## Abstract

**Background:**

Interoception plays a vital role in human cognition and emotion and is an increasingly important part of clinical studies of mind–body approaches and mental health. Interoceptive awareness (IA) encompasses numerous mind–body components and can be assessed by employing a self-report measure such as the Multidimensional Assessment of Interoceptive Awareness (MAIA), which has been adapted and validated across several countries and is used in experimental and clinical settings. In this study, the MAIA-2, which was developed due to the psychometric shortages of MAIA, was thoroughly translated, and its psychometric features were examined in a sample of 306 Norwegian-speaking participants (81% females, ages 16 through 66 plus).

**Methods:**

The participants completed the MAIA-2 Norwegian version (MAIA-2-N) and the COOP/WONCA Functional Assessment Charts measuring psychological, physical, and overall health. The following psychometric qualities of the MAIA-2 were investigated: factor structure, internal consistency, and the moderating role of gender.

**Results:**

Confirmatory Factor Analysis (CFA) revealed that an 8-factor model of MAIA-2-N provided the best fit. Also, a bifactor model revealed a proper fit. Good internal consistency and a moderating role of gender, age, and education on the relationships between certain MAIA-2-N factors and health were observed.

**Conclusions:**

The MAIA-2-N is an adequate measure of IA in Norwegian-speaking individuals. The factor-structure corresponds with the original MAIA-2 and it shows good internal consistency. Some moderating effects of gender were observed, particularly related to the relationship between IA and physical and psychological state, with the physical state/fitness more closely linked to IA in males and psychological state in females.

## Key Notes:

- The MAIA-2 aims to measure Interoceptive Awareness (IA) through self-report.
- Psychometric properties of the Norwegian version of MAIA-2 (MAIA-2-N) were explored, showing the same factor structure as the original MAIA-2 along with good internal consistency.
- Gender played a moderating role between IA components and psychological, physical, and overall health states.

## Introduction

Interoception refers to the sensing of our internal state, which can include changes in heart rate, the distention of the gut, internal temperature, hydration levels, information coming from free nerve endings in the fascia and muscles, as well as hormones, stretch, and pain receptors [1]. Interoception is profoundly entwined with affect and motivation directly related to the homeostatic state of the body and is vital to our sense of self, consciousness, and health [2–5], including mental health [6]. Affective and motivational states can be seen as arising from interpretations of and changes in interoceptive signals [5, 7]. For instance, physiological conditions such as dehydration or the buildup of carbon dioxide in the blood can cause feelings of anxiety through interoception [8]. Adequate interoceptive awareness (IA), sensitivity, and accuracy are important for self-regulation, allowing the brain to make homeostatic predictions of current and prospective needs and take action to meet those needs (for instance, by rest or intake of fluids) [5, 9]. Low interoceptive sensitivity or awareness can, therefore, make internal states unclear and harder to manage. However, too much interoceptive sensitivity or awareness may also be detrimental, as high interoception may lead to overwhelming or intrusive sensations with little adaptive value [10]. There is, therefore, an adaptive spectrum of interoceptive ability ranging from the *hypo*-aware/sensitive to the *hyper*-aware/sensitive.

In line with the importance of interoception for adaptive self-regulation (e.g., [9, 10]) recent theories of psychopathology state that a lack of access to valid, consistent, or reliable information about inner state (i.e., impaired or disturbed interoception and lack of body-brain integration) or distortions in how we interpret these interoceptive signals can lead to extensive difficulties with adaptive regulation, including anxiety and depression [5, 6, 9]. Disorders like anxiety or depression [11], sleep disorders [12], obsessive–compulsive disorder [13], eating disorders [14], addiction [15], certain physical conditions [3], and even difficulties with social interactions [16] can within such a perspective be understood as a disturbance in the ability to process and integrate interoceptive information giving an impoverished basis for adaptive predictions and fundamental self-regulation. In line with this, most mental disorders are characterized by varying problems with autonomic dysfunctions and emotion dysregulation [17–19], and disturbed interoception is associated with various mental disorders (e.g., [6]) as well as an increased risk of developing psychopathology prospectively [20, 21]. Furthermore, recent research suggests interventions such as interoceptive training may alleviate anxiety and depression and improve function [22–24]. Thus, research on different aspects of interoception, including interoceptive awareness, is important for understanding mental health. This necessitates access to psychometrically sound and validated measures and knowledge about possible moderating factors, such as gender.

### Gender and interoceptive awareness

There is a well-known gender gap in risk for psychopathology, with females showing a higher risk of developing mental disorders from puberty onwards [25]. While the gender gap likely has many complex contributing factors, the higher risk of psychopathology in females may at least be partially linked to atypical interoception coupled with physical changes across the lifespan [26]. Previous research has shown gender differences in interoception, with females scoring higher on dimensions related to interoceptive and emotional awareness and males showing less worry and more trust related to their bodily experiences [27]. The same study also revealed gender differences in interoceptive accuracy. Gender differences in interoception have been suggested as a possible contributing factor to the increased risk of psychopathology in females, especially around transitional times involving major physical changes such as adolescence, pregnancy, or menopause [26]. In particular, the tendency for females to show higher interoceptive attention coupled with lower interoceptive accuracy [27, 28] could lead to both less adaptive self-regulation and increased psychological distress due to higher interoceptive prediction error rates, particularly at times of pronounced physiological change.

### The MAIA and pathways to MAIA-2

Interoception can be measured using psychophysiological measures with varying degrees of accuracy, validity, and invasiveness [6, 29]. Although arguably less objective and with some concerns related to a lack of concept-convergence [30], self-report measures have a use in the study of interoception, mainly due to ease of use, non-invasiveness, and access to the experiential world of participants. A widely used questionnaire for assessing interoceptive awareness is the Multidimensional Assessment of Interoceptive Awareness (MAIA, [31]), which measures IA using 32 items across eight factors. The factor structure of MAIA and the factor’s conceptual contents are as follows: *Noticing* (the ability to be aware of uncomfortable, comfortable, and neutral body sensations), *Not-Distracting* (the tendency not to ignore or distract oneself from sensations of pain or discomfort), *Not-Worrying* (the tendency not to worry or experience emotional distress related to sensations of pain or discomfort), *Attention Regulation* (the ability to sustain and control attention to body sensations), *Emotional Awareness* (awareness of the connection between body sensations and emotional states), *Self-Regulation* (the ability to regulate distress by directing attention to body sensations), *Body Listening* (the tendency to actively listen to the body for insight), and finally *Trusting* (the experience of one's body as safe and trustworthy). MAIA has been adapted and employed in a variety of countries, and its psychometric properties have been investigated previously [32–34]. Due to reported low internal consistency for two of the factors (Not-Distracting and Not-Worrying) across several psychometric studies of MAIA (e.g., [34, 35]), a second version (MAIA-2) was developed and validated in 2018 [36]. Five items were added to the Not-Distracting and Not-Worrying subscales, bringing the total count of MAIA-2 to 37 items. Validation of MAIA-2 in 1090 participants found that the internal consistency improved in the two problem-scales in MAIA while confirming the same 8-factor structure of the original MAIA, and it is now recommended that future studies use MAIA-2 [35, 36]. To the best of our knowledge, only two further studies have investigated the psychometric features of the MAIA-2 thus far. Eggart and colleagues [37] investigated the psychometric properties of MAIA-2 in a German clinically depressed sample, while Özpinar and colleagues [38] tested it in a Turkish sample (See Table 1). Few studies have focused on gender differences in IA using MAIA thus far; however, earlier research indicates invariance across gender [39]. Further investigations of gender differences in interoception and specifically in MAIA-2 are, therefore, warranted.

**Table 1:** Examinations of the factorial structure of the MAIA-2

Reference	Language	Country	Sample type	N	Data reduction method	Dimensionality	Fit Indices (Final Model)	Cronbach α values
(Mehling et al., 2018) [36]	English	United Kingdom	Community (57% females; Age: 18 to 69)	1090	EFA and CFA	Eight dimensions (37 items)	χ2(601) = 1597.7 *p* < .0001; RMSEA = .055; CFI > .90;	*N* = .64 ND = .74 NW = .67 AR = .83 EA = .79 SR = .79 BL = .80 TR = .83
(Özpinar et al., 2021 [38])	Turkish	Turkey	Health care staff (54.7% females; Age: 46.85 (11.23))	400	EFA and CFA	Six Dimensions (37 items)	χ2 = 5134.120, p < 0.001 CFI = 1; RMSEA = .00	EA = 1 AR = .85 BL = 1 ND = .92 TR = .63 NW = .998
(Eggart et al., 2021) [22, 37]	German	Germany	Depressed individuals (female: 55.45%)_	110		Eight dimensions (37 items)	Not Specified	N = .64 ND = .67 NW = .71 AR = .85 EA = .86 SR = .74 BL = .75 TR = .85

*N* Noticing, *NT* Not-Distracting, *NW* Not-Worrying, *AR* Attention Regulation, *EA* Emotional Awareness, *ER* Self- Regulation, *BL* Body Listening, *TR* Trusting, *CFI* Comparative Fit Index, *RMSEA* Steiger-Lind root mean square error of approximation, *EFA* Exploratory Factor Analysis, *CFA* Confirmatory Factor Analysis

### Aims of the current study

The current study used Confirmatory Factor Analysis (CFA) to investigate the factor structure of MAIA-2-N, specifically comparing an 8-factor solution proposed by Mehling et al. [36] in the original study and a 6-factor solution posited in the Turkish adaptation [38], with a sample of Norwegian-speaking participants. Also, according to a recent review, there may be possible gender differences in IA [40]. Therefore, we investigated potential gender differences in interoception. This included the moderating role of gender on the relationships between IA and COOP/WONCA subscales and age and education. Based on the literature, which shows linkages between IA and psychiatric conditions such as heightened anxiety, emotion dysregulation, alexithymia (e.g., [31, 32]), and physical health conditions (e.g., [3]), we also investigated the relationships between IA subscales and measures of subjectively experienced daily and social activity, change in health, psychological distress, and overall health. Finally, a one-bifactor model was examined as a competitive model for both one global-factor and 8-factor orthogonal models demonstrated in previous investigations. The bifactor framework was devised to integrate construct-relevant multidimensionality in order to conduct a more detailed psychometric analysis of multifaceted measures [41].

## Method

### Participants

Participants were 306 Norwegian speakers (19% males and about 0.65% with no specified gender) recruited from two Norwegian municipalities and two Norwegian Universities as well as online recruitment. The invitation to participate called for fluent Norwegian speakers. Norwegian proficiency was not tested, but all study information and all questions, including the invitation to participate were in Norwegian. Age was assessed in 11 age brackets from 16–20 (1.4%), 21–25(8.2%), 26–30(14.1%), 31–35(12.7%), 36–40(11.1%), 41–45(17.6%), 46–50(15.7%), 51–55(10.5%), 56–60(2.9%), 61–65(4.6%) and 66 years and over (1.3%). All age brackets were represented, with the median age reported as 41- 45 years (17.6% of the sample). Education was assessed as the highest completed level of education at the time of the survey using a 3-point scale ranging from 1) completed high-school/vocational school or lower, 2) bachelor's degree, or 3) master's degree/Ph.D. graduate. Of the full sample, 42 participants (13.7%) listed high school/vocational school as their highest completed degree, 100 participants reported having completed a bachelor's degree (32.7%), while 164 participants reported finishing a master's degree or above (53.6%).

### Measures

#### The Multidimensional Assessment of Interoceptive Awareness-2 (MAIA 2)

MAIA-2 has 37 items that are answered on a 6-point Likert scale ranging from 0 "*never*" to 5 "*always*", with nine reverse-scored items. In the original version of MAIA-2, *Cronbach alphas* for the eight scales ranged from 0.64 to 0.83 [36]. Likewise, in another study of MAIA-2 among depressed German-speaking individuals, reliability was deemed sufficient at *ω* = 0.70- 0.90 [37]. Reliability for the 6-factor Turkish version for all subscales was over 0.60 in a Turkish adaption [38]. The current study used a new Norwegian translation of the original MAIA-2 (MAIA-2-N) conducted by two of the authors.

#### Dartmouth Coop Functional Health Assessment/World Organization of National Colleges, Academies and Academic Association of General Practitioners (COOP/WONCA)

Physical state/fitness and psychological/emotional state and overall health were assessed with the COOP/WONCA [42, 43]. COOP/WONCA consists of five charts that describe various aspects of health status: overall health (*How would you rate your health in general?*), physical state/fitness (*What was the hardest physical activity you could do for at least 2 min?)*, feelings^1^ (*How much have you been bothered by emotional problems such as feeling anxious, depressed, irritable or downhearted and sad?*), change in health (*How would you rate your overall health now compared to 2 weeks ago?)* and daily activities (*How much difficulty have you had doing your usual activities or tasks, both inside and outside the house because of your physical and emotional health?*) and social activities (*Has your physical and emotional health limited your social activities with family, friends, neighbors or groups?*) during the past two weeks. The five areas are scored individually on a scale from 1–5 (where one is the least problematic). The questionnaire has been previously tested in Norway with acceptable results [44], showing inter-rater reliability Kappas for physical state/fitness at K = 0.59, psychological state K = 0.58, and overall health at K = 0.65 [44]. For ease of interpretation, the scores on the COOP/WONKA were reversed for the statistical analyses so that a higher score on each of the dimensions indicates a positive state (i.e., better self-perceived function/state/health).

### Procedure

#### Translation of the MAIA-2

The translation followed World Health Organization (WHO)’s recommendations [45], with two independent translators performing separate translations before discussing and settling any differences in the translations. The Norwegian-translated version was further discussed with two experts in psychosomatic clinical work and long experience with patient groups with reduced interoception. Based on this discussion, a further effort was made to simplify the language without losing meaningful content or altering the meaning of items, to make the instrument more suitable for use in clinical populations where language proficiency may be lower and clear communication is of importance [46]. After the translators and clinical experts agreed on a final version, a third, independent back-translator fluent in Norwegian and English and with an understanding of psychology and interoception back-translated the items and instructions into English. The back-translated version was highly consistent with the original MAIA-2.

#### Ethical considerations

All participants were given written information about the study and gave informed consent before participating. The study complied with the Helsinki Declaration [47].

#### Design and recruitment

The study took place as an anonymous online survey. The survey called for subjects > 16 years in the general population who could read and understand Norwegian well. Participants were recruited among employees from two Norwegian municipalities (one urban and one rural), employees and students at two Norwegian universities, employees at the local university hospital, and through information in social media. The data was collected using a secure internet-survey solution (Nettskjema) and took approximately 20 min to complete. The participants were asked to answer demographic questions about gender, age, and education before answering the questions in the MAIA-2-N. Finally, the participants were asked questions about their general self-perceived physical state/fitness and psychological state, and overall health and daily function. With no data loss, 306 individuals in total responded.

#### Analysis strategy

The data screening was performed using IBM SPSS Statistics (Version 28). Thus, list-wise deletion with no data imputation was considered in the current analyses [48]. The normality assumption was tested, and skewness was calculated. First, to conduct the Confirmatory Factor Analysis, Mplus 8.8 version [49] was utilized to determine the factorial structure of the MAIA-2-N, and Goodness of Fit was tested for four models. We applied the CFA using the weighted least square mean and variance adjusted (WLSMV) estimator to examine a priori models of the factor structure proposed by Mehling [36] and Özpinar [38]. In model 1, which is a one-factor model, all 37 items were made to load on a single factor of general IA [36]. Model 2 describes a 6-factor oblique model [38]. Model 3 evaluates an 8-factor orthogonal model, and Model 4 estimates an 8-factor oblique model, as reported by Mehling [36]. Model 5 is an 8-factor first-order and one-factor second-order model. In the higher-order model, more than one orthogonal first-order subordinate factor mediates the relationship between observed indicators and superordinate second-order latent factors [50]. The largely standardized covariances (-1 ≤ r ≤ 1) among latent factors in the 8-factor oblique model indicate that more than the first-order model is needed to account for the estimated variances and covariances of eight IA subscales. Model 5 was then tested to see whether there might be a common general IA factor that underlies all eight IA domains. Finally, Model 6 evaluated a hypothesized bifactor model in which all items loaded on specific 8-factor first-order orthogonal subscales and an overarching first-order general trait. A bifactor model is essential in assessing factor structure and applying the total raw scores for multi-dimensional scales [51].

To investigate the MAIA-2-N stability, CFA provides a variety of statistical tests for measuring the "Goodness-of-Fit" of the identified models used [52–55]. The statistics that were chosen a priori for this study were the Comparative Fit Index (CFI > 0.95), normal Chi-square (*χ2/df* < 3), the Root Mean Square Error of Approximation (RMSEA), and its 90% confidence interval < 0.06., the Chi-square (*χ*^*2*^; desired p > 0.05), the Tucker–Lewis Index (TLI > 0.95), and the Standardized Root Mean Square Residual (SRMR < 0.06). Since there was a multivariate skewness in the data, the fit indices of all models were corrected with the Satorra-Bentler scaled difference Chi-square test statistic [56]. The fitted models were nested; in these cases, the comparative fit was investigated by *χ*^*2*^ difference tests (^*2*^) and the interpretability of the solutions.

Second, as it is recommended for ordinal Likert-type scales, the internal consistency was examined using Cronbach's alpha, mean inter-item correlation, and the equivalent of Cronbach's alpha coefficient (ordinal alpha and omega reliability), which are based on the polychoric correlation, rather than the Pearson correlation [57, 58]. This calculation was conducted in R version 4.1.2 [59, 60]. According to a rule of thumb, a correlation coefficient of 0.70 or higher was considered an acceptable level of internal consistency of the items [61].

Third, an independent t-test and multivariate analysis of variances (MANOVA) were conducted to investigate the gender-based difference between males and females on the mean scores of MAIA-2-N and its subscales (as dependent variables), with gender used as an independent variable in the analysis [62].

Fourth, due to evidence of non-normality in the data, the relationships between the MAIA-2-N scores, physical state/fitness, psychological state, overall health, social and daily activities and change in health, age groups (ordered variable), and educational level (ranked variable) were investigated using Spearman correlations. Given the number of correlations, the *p* values were set at 0.05 to control for the experiment-wise error. The correlation coefficients are interpreted as follows: correlations of 0.10 are considered weak, 0.20 are considered moderate, and above 0.30 are fairly strong based on studies of typical effect sizes in psychological research [63, 64].

Fifth, in an examination of the relationships between interoceptive awareness and physical state/fitness and psychological state, overall health, age, and educational level, a Fisher's r-to-z approach [65–67] was used to explore the moderating role of gender.

## Results

### Interoceptive awareness factor structure

**Fig. 1:**
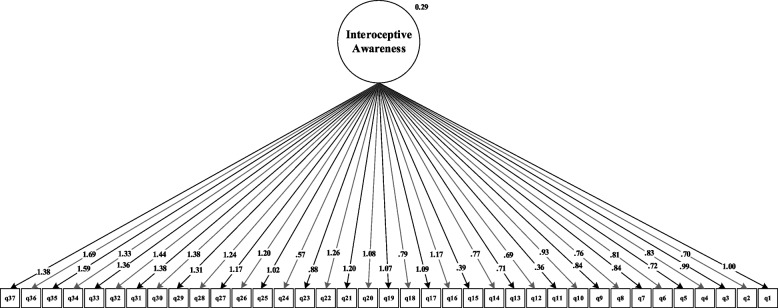
Model one: the one-factor oblique and correlated errors model of the MAIA-2-N. Notes. MAIA-2-*N* = Multidimensional assessment of interoceptive awareness-2 Norwegian version. Fit indices: *χ2* = 5740.302, df = 629, CFI = .597, RMSEA = .163, SRMR = .134

**Fig. 2:**
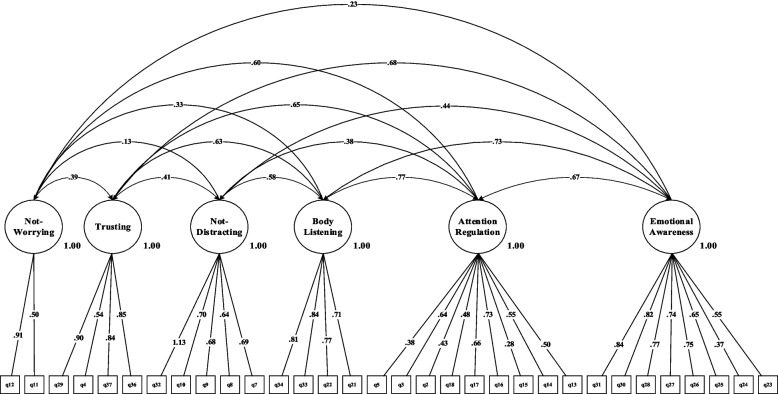
Model two: the six-factor model of the MAIA-2-N. Notes. MAIA-2-N = the Multidimensional assessment of interoceptive awareness-2 Norwegian version. Fit indices: *χ2* = 3482.585, df = 449, CFI = .691, RMSEA = .149, SRMR = .120

**Fig. 3:**
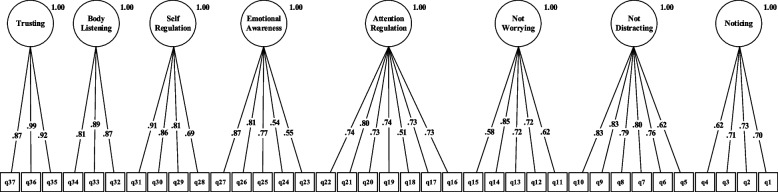
Model three: the eight-factor orthogonal model of the MAIA-2-N. Notes. MAIA-2-N = the Multidimensional assessment of interoceptive awareness-2 Norwegian version. Fit indices: *χ2* = 5689., df = 628, CFI = .600, RMSEA = .162, SRMR = .216

**Fig. 4:**
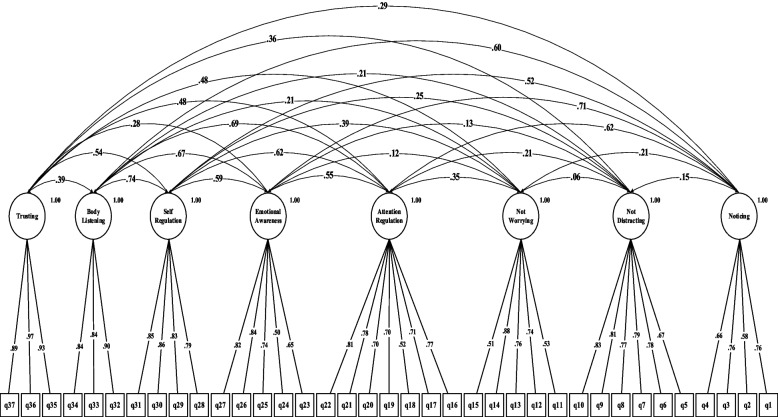
Model four: the eight-factor oblique model of the MAIA-2-N. Notes. MAIA-2-N = the Multidimensional assessment of interoceptive awareness-2 Norwegian version. Fit indices: *χ2* = 1223.737, df = 601, CFI = .951, RMSEA = .162, SRMR = .216

**Fig. 5:**
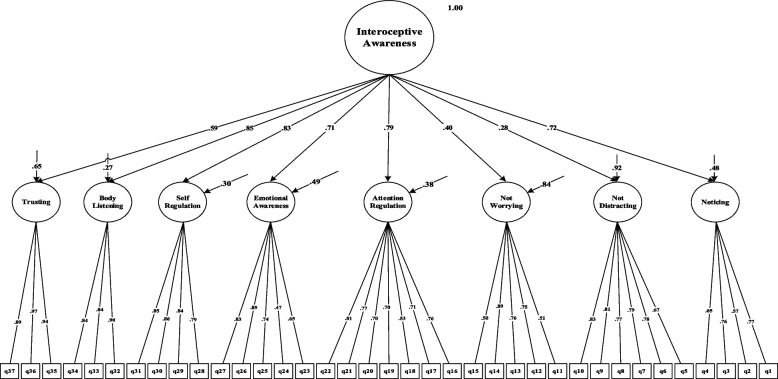
Model five: the eight-factor first-order and one-factor second-order model of the MAIA-2-N. Notes. MAIA-2-N = the Multidimensional assessment of interoceptive awareness-2 Norwegian version. Fit indices: *χ2* = 1481.034, df = 621, CFI = .932, RMSEA = .067, SRMR = .071

**Fig. 6:**
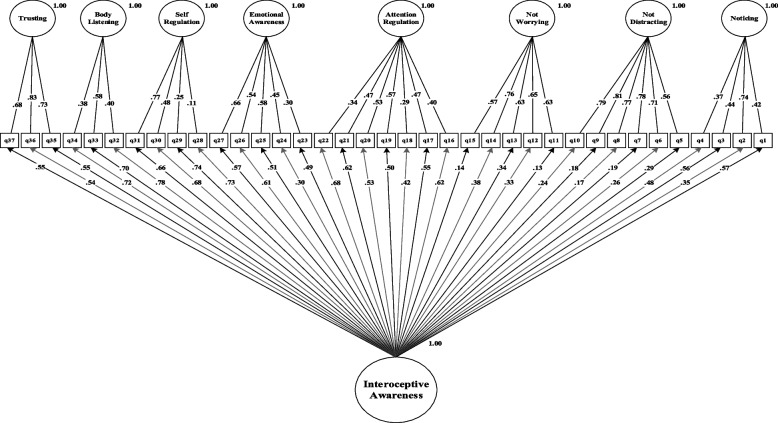
Model six: the bifactor model of the MAIA-2-N. Notes. MAIA-2-N = the Multidimensional assessment of interoceptive awareness-2 Norwegian version. Fit indices: *χ2* = 1431.342, df = 592, CFI = .934, RMSEA = .068, SRMR = .066

The results of the fit estimates for all models are presented in Table 2 and Figs. 1, 2, 3, 4, 5 and 6. The one-factor/general, 6-factor oblique (proposed by the Turkish version), and the 8-factor orthogonal models did not meet the previously specified fit criteria (i.e., *S-Bχ*^*2*^*/df* < 3, CFI > *0.95,* TLI > *0.95, RMSEA* < 0.06) while the 8-factor oblique model revealed adequate fit to the data (for more details see Table 2; M_1_ to M_6_ and Figs. 1, 2, 3, 4, 5 and 6). The fitness of the 8-factor oblique and 6-factor oblique models were compared using the parsimony principle (Table 2; M_2_ & M_4_: *ΔS-Bχ*^*2*^ = 2258.84, *Δdf* = 152, *p* < 0.001).

**Table 2:** Internal consistency coefficients and parameter estimates and goodness-of-fit indexes for CFA of the MAIA-2-N

	Items (Original in English, copyright Mehling, 2018. Found at http://www.osher.ucsf.edu/maia/)						r^cs^	r^ct^	α	Ordinal Alpha	Omega
Noticing	1. Når jeg er anspent legger jeg merke til hvor spenningen sitter i kroppen min. (When I am tense I notice where the tension is located in my body.)						.57	.55	.75	.78	.75
	2. Jeg legger merke til når jeg er ukomfortabel i kroppen min. (I notice when I am uncomfortable in my body.)						.56	.33			
	3. Jeg legger merke til hvor jeg er komfortabel i kroppen min. (I notice where in my body I am comfortable.)						.56	.51			
	4. Jeg legger merke til endringer i pusten min, sånn som at den går saktere eller raskere. (I notice changes in my breathing, such as whether it slows down or speeds up.)						.50	.42			
Not-Distracting	5. Jeg overser fysisk anspenthet eller ubehag til det blir mer alvorlig. (I ignore physical tension or discomfort until they become more severe.)						.54	.35	.87	.89	.87
	6. Jeg distraherer meg selv fra følelser av ubehag. (I distract myself from sensations of discomfort.)						.69	.35			
	7. Når jeg føler smerte eller ubehag, prøver jeg å bare kjøre på / kjempe meg igjennom det. (When I feel pain or discomfort, I try to power through it.)						.69	.29			
	8. Jeg prøver å overse smerte. (I try to ignore pain.)						.68	.26			
	9. Jeg skyver bort følelser av ubehag ved å fokusere på noe. (I push feelings of discomfort away by focusing on something.)						.72	.31			
	10. Når jeg kjenner ubehag i kroppen opptar jeg meg med noe annet, så jeg ikke trenger å kjenne det. (When I feel unpleasant body sensations, I occupy myself with something else so I don’t have to feel them.)						.71	.34			
Not-Worrying	11. Når jeg føler fysisk smerte, blir jeg ute av meg. (When I feel physical pain, I become upset.)						.53	.17	.78	.82	.78
	12. Jeg begynner å bekymre meg for at noe er galt hvis jeg føler ubehag. (I start to worry that something is wrong if I feel any discomfort.)						.61	.35			
	13. Jeg kan legge merke til en ubehagelig fornemmelse/følelse i kroppen uten å bekymre meg for den. (I can notice an unpleasant body sensation without worrying about it.)						.54	.32			
	14. Jeg kan holde meg rolig og ikke bekymre meg når jeg føler ubehag eller smerte. (I can stay calm and not worry when I have feelings of discomfort or pain.)						.64	.35			
	15. Når jeg har ubehag eller smerter, klarer jeg ikke få det ut av hodet. (When I am in discomfort or pain I can’t get it out of my mind.)						.48	.18			
Attention Regulation	16. Jeg kan være oppmerksom på pusten min uten å bli forstyrret av ting som skjer rundt meg. (I can pay attention to my breath without being distracted by things happening around me.)						.62	.57	.85	.87	.85
	17. Selv når det skjer mye rundt meg kan jeg holde oppmerksomheten på det jeg kjenner i kroppen min. (I can maintain awareness of my inner bodily sensations even when there is a lot going on around me.)						.65	.52			
	18. Jeg kan følge med på kroppsholdningen min når jeg er i samtale med noen. (When I am in conversation with someone, I can pay attention to my posture.)						.46	.39			
	19. Jeg kan snu oppmerksomheten tilbake til kroppen min hvis jeg blir distrahert/forstyrret. (I can return awareness to my body if I am distracted.)						.64	.52			
	20. Jeg kan snu oppmerksomheten min fra å tenke til å kjenne kroppen min. (I can refocus my attention from thinking to sensing my body.)						.63	.53			
	21. Jeg kan fortsette å være oppmerksom på hele kroppen min selv om en del av meg har smerter eller ubehag. (I can maintain awareness of my whole body even when a part of me is in pain or discomfort.)						.67	.59			
	22. Jeg klarer å bevisst fokusere på kroppen min som en helhet. (I am able to consciously focus on my body as a whole.)						.59	.63			
Emotional Awareness	23. Jeg legger merke til hvordan kroppen min endrer seg når jeg er sint. (I notice how my body changes when I am angry.)						.50	.46	.80	.83	.81
	24. Når noe er galt i livet mitt kan jeg kjenne det i kroppen min. (When something is wrong in my life I can feel it in my body.)						.45	.28			
	25. Jeg legger merke til at kroppen min føles annerledes etter en fredelig opplevelse. (I notice that my body feels different after a peaceful experience.)						.67	.45			
	26. Jeg legger merke til at pusten min blir fri og lett når jeg føler meg komfortabel. (I notice that my breathing becomes free and easy when I feel comfortable.)						.63	.56			
	27. Jeg legger merke til hvordan kroppen min endrer seg når jeg føler meg glad / lykkelig. (I notice how my body changes when I feel happy / joyful.)						.70	.54			
Self-Regulation	28. Når jeg føler meg overveldet / ting blir for mye for meg kan jeg finne et rolig sted inni meg. (When I feel overwhelmed, I can find a calm place inside.)						.59	.64	.85	.87	.86
	29. Når jeg flytter oppmerksomheten til kroppen min kjenner jeg en følelse av ro. (When I bring awareness to my body, I feel a sense of calm.)						.72	.64			
	30. Jeg kan bruke pusten min til å redusere spenninger. (I can use my breath to reduce tension.)						.69	.61			
	31. Når jeg er opphengt i tanker kan jeg roe hodet mitt ved å fokusere på kroppen eller pusten min. (When I am caught up in thoughts, I can calm my mind by focusing on my body/breathing.)						.77	.61			
Body Listening	32. Jeg lytter til / kjenner etter i kroppen min for å finne ut hva jeg føler. (I listen for information from my body about my emotional state.)						.75	.68	.87	.89	.93
	33. Når jeg er opprørt tar jeg meg tid til å utforske hvordan kroppen min kjennes ut. (When I am upset, I take time to explore how my body feels.)						.77	.63			
	34. Jeg lytter til / kjenner etter i kroppen min så den kan fortelle meg hva jeg skal gjøre. (I listen to my body to inform me about what to do.)						.72	.60			
Trusting	35. Jeg føler meg hjemme i kroppen min. (I am at home in my body.)						.86	.80	.93	.95	.93
	36. Jeg føler at kroppen min er et trygt sted. (I feel my body is a safe place.)						.90	.84			
	37. Jeg stoler på det jeg kjenner i kroppen min. (I trust my body sensations.)						.81	.73			
_Model_	_*χ*_^*2*^	_*df*_	_*χ*_^*2*^_/df_	_CFI_	_TLI_	_RMSEA_	_SRMR_	_Base_	_ΔS-Bχ_^2^_(Δdf)_		
M_1=_ one-factor model	5740.302	629	9.12	.597	.573	.163(.159-.167)	.134	-	-		
M_2=_ 6-factor oblique model	3482.585	449	7.75	.691	.658	.149(.144-.153)	.120	M_1_	2257.71^**^(177)		
M_3=_ 8-factor orthogonal model	5689.240	628	9.06	.600	.576	.162(.158-.166)	.216	M_1_	51.06^**^(1)		
M_4=_ 8-factor oblique	1223.737	601	2.03	.951	.946	.058(.054-.063)	.055	M_1_	4516.57^**^(28)		
M_5=_ 8-factor first-order and one-factor second-order model	1481.034	621	2.38	.932	.927	.067(.063-.072)	.071	M_1_	4259.27^**^(8)		
M_6=_ bifactor model	1431.342	592	2.42	.934	.925	.068(.064-.073)	.066	M_1_	4308.96^**^(37)		

*MAIA-2-N* the Multidimensional Assessment of Interoceptive Awareness-2 Norwegian version. *r*^*cs*^ Corrected item-total correlation for subscales’ items, *r*^*ct*^ Corrected item-total correlation for scales’ items, α Cronbach`s alpha, *χ*^*2*^ Chi-square, *df* Degrees of freedom, *χ*^*2*^/df Normal chi-square, *TLI* Tucker–Lewis index, *CFI* Comparative fit index, criterion, *SRMR* Standardized root mean square residual, *RMSEA* Root mean square error of approximation, Δ*χ*^*2*^ Difference between minus twice log likelihoods between the full and the nested models, ^∗^*p* < .05, ^∗∗^*p* < .01, ^∗∗∗^*p* < .001

As depicted in Table 2, the evaluation of the bi-factor orthogonal model according to the previously specified fit criteria (M_6_: *S-Bχ*^*2*^*/df* = 2.42, CFI > *0.93,* TLI > *0.92, RMSEA* < 0.068) was acceptable. Then, the principle of parsimony [68] was used to compare the fit indices of the 8-factor first-order and one-factor second-order model (M_5_) and bifactor model (M_6_) as nested models with those of the M_1_ as the baseline/null model. Finally, fit indices of M_5_ with M_4_ (*S-Bχ*^*2*^ = 257.30, *df* = 20, *p* < 0.001), M_6_ with M_4_ (*S-Bχ*^*2*^ = 207.61, *df* = 9, *p* < 0.001), and M_6_ with M_5_ (*S-Bχ*^*2*^ = 49.69, *df* = 29, *p* < 0.05) were compared as competitive models to get an optimal/parsimonious model.

As indicated in Table 2 and Figs. 1, 2, 3, 4, 5 and 6, none of the models met most of the specified fit criteria, except for the 8-factor oblique model (M4; i.e., theory-derived model; [36]). Therefore, the 8-factor model met most of the specified fit criteria and provided a better fit (M_4;_ *S-Bχ*^*2*^*/df* = 2.03; CFI = 0.95; TLI = 0.95; and RMSEA = 0.056; [CI] 90% = 0.054, 0.063).

### Internal consistency

In Table 2, internal consistency coefficients and corrected item-total correlation for items of the MAIA-2-N have been presented. The means of inter-item correlation were 0.45, 0.53, 0.42, 0.45, 0.45, 0.59, 0.68, and 0.81 for Noticing, Not-Distracting, Not-Worrying, Attention Regulation, Emotional Awareness, Self-Regulation, Body Listening, and Trusting, respectively. Cronbach’s alpha, ordinal alpha, and omega coefficients for the subscales of MAIA-2-N ranged from 0.75 to 0.93; 0.78 to 0.95, and 0.75 to 0.93, respectively.

### Interoceptive awareness and gender

Table 3 presents the means and standard deviations of the Multidimensional Assessment of Interoceptive Awareness-2-Norwegian (MAIA-2-N), the COOP/WONCA, and their respective subscales across gender. Prior to investigating gender differences in the mean scores of the MAIA-2-N, multivariate analysis of variances (MANOVA) was conducted to ensure the homogeneity of groups by examining health-related variables between males and females. Subsequently, significant subscales of the COOP/WONCA were considered covariate variables in the assessment of interoceptive awareness and gender differences. Hence, the main effect of physical state/fitness [*F* (1, 302) = 14.43, *p* < 0.001, *η*^*2*^ = 0.05] and daily activity [*F* (1, 302) = 7.49, *p* = 0.007, *η*^*2*^ = 0.03] was controlled in testing the gender effect on MAIA-2-N mean scores.

**Table 3:** Means, standard deviations, and correlation coefficients of the MAIA-2-N in males (*n* = 57) and females (*n* = 247)

	1	2	3	4	5	6	7	8	9	Mean (SD)		
										female	male	total
1. N	-	.12^*^	.12^*^	.52^**^	.55^**^	.42^**^	.49^**^	.22^**^	.65^**^	13.06(3.47)	12.51(4.02)	12.98(3.58)
2. ND		-	.08	.19^**^	.11	.22^**^	.19^**^	.33^**^	.50^**^	12.41(5.36)	11.89 (5.32)	12.33(5.34)
3. NW			-	.26^**^	.03	.31^**^	.16^**^	.40^**^	44^**^	14.70(3.96)	16.42(3.61)	15.03(3.94)
4. AR				-	.44^**^	.57^**^	.62^**^	.42^**^	.79^**^	18.89(5.66)	19.30(6.30)	18.99 (5.77)
5. EA					-	.46^**^	.55^**^	.21^**^	.63^**^	14.42(3.39)	12.86(3.74)	14.13(3.50)
6. SR						-	.67^**^	.49^**^	.79^**^	10.02(3.97)	9.46(4.83)	9.94(4.15)
7. BL							-	.35^**^	.76^**^	6.69(3.28)	5.82(3.47)	6.56(3.35)
8. TR								-	.65^**^	9.95(3.90)	11.28(3.06)	10.22(3.78)
9. MAIA-2-N	.65**	.50**	.44**	.79**	.63**	.79**	.76**	.65**	-	103.46(21.97)	102.64(24.05)	103.46(22.34)
10. PhF	.10	.18^**^	.19^**^	.10	.08	.06	.01	.31^**^	.20^**^	1.94(.90)	1.46(.68)	1.85(.88)
11. PS	.15^**^	.27^**^	.34^**^	.28^**^	.04	.38^**^	.16^**^	.56^**^	.42^**^	2.59(1.02)	2.47(1.12)	2.57(1.04)
12. OH	.18^**^	.28^**^	.30^**^	.31^**^	.18^**^	.37^**^	.23^**^	.55^**^	.46^**^	2.35(.86)	2.14(.83)	2.31(.86)
13. ChH	.04	.11	.01	.04	.09	.07	.05	.07	.09	2.82(.67)	2.96(.60)	2.85(.65)
14. DA	.10	.22**	.29**	.20**	.04	.19**	.08	.42**	.30**	1.88(.99)	1.49(.85)	1.81(.98)
15. SA	.04	.22**	.27**	.17**	.02	.17**	.06	.40**	.26**	1.79(1.03)	1.60(.94)	1.75(1.01)
16. Age	.06	-.11*	.08	.06	.05	.21**	.09	.07	.09			
17. Education	.03	.00	.06	.08	.05	.12*	.12*	.12*	.01			
18. PhFAG	(.12, .05)	(.19^**^, .19)	(.13^*^, .43^**^)	(.08, .13)	(.07, .17)	(.05, .11)	(.04,.09)	(.26^**^, .23)	(.17^**^, .19)			
19. FsG	(.19^**^, .01)	(.27^**^, .24)	(.37^**^, .03)	(.33^**^, .06)	(.06, .05)	(.43^**^, .12)	(.20^**^, .02)	(.56^**^, .52^**^)	(.47^**^, .15)			
20. OHG	(.15^*^, .15)	(.25^**^, .40^**^)	(.30^**^, .14)	(.27^**^, .36^**^)	(.13^*^, .44^**^)	(.35^**^, .39^**^)	(.21^**^, .33^**^)	(.54^**^, .53^**^)	(.41^**^, .49^**^)			
21. CHG	(.04, .03)	(.12, .03	(.03, .09)	(.01, .02)	(.09, .03)	(.06, .02)	(.04, .09)	(.05, .01)	(.08, .02)			
22. DAG	(.09, .01)	(.19^**^, .29^*^)	(.25^*^, .32^*^)	(.21^**^, .18)	(.08, .02)	(.22^**^, .17)	(.12, .09)	(.34^**^, .56^**^)	(.28^**^, .27^*^)			
23. SAG	(.03, .10)	(.22^**^, .20)	(.25^**^, .29^*^)	(.19^**^, .18)	(.01, .16)	(.19^**^, .18)	(.09, .01)	(.34^**^, .56^**^)	(.25^**^, .27^*^)			
24. AgeG	(.05, .07)	(-.10, -.28^*^)	(.12, -.03)	(.09, -.06)	(.06, -.06)	(.23^**^, .13)	(.10, .04)	(.13^*^, -.21)	(.13^*^, -.07)			
25. EduG	(.04, .10)	(.04, -.03)	(.12, -.06)	(.09, -.05)	(.10, -.12)	(.17^**^, -.11)	(.15^*^, -.07)	(.18^*^, -.20)	(.14^*^, -.10)			

*MAIA-2-N* the Multidimensional Assessment of Interoceptive Awareness-2 Norwegian Version. ∗ *p* < .05, ∗  ∗ *p* < .01, ∗  ∗  ∗ *p* < .001. *N* Noticing, *NT* Not Distracting, *AR* Attention Regulation, *EA* Emotional Awareness, *ER* Self- Regulation, *BL* Body Listening, *TR* Trust, *PhF* Physical State/Fitness, *PS* Psychological State, *OH* Overall Health, *ChH* Change in Health, *DA* Daily Activity, *SA* Social Activity, *PhFG* Physical State/Fitness across Gender, *PSG* Psychological State Across Gender, *OHG* Overall health state across Gender, *CHG* Change in Health across Gender, *DAG* Daily Activity across Gender, *SAG* Social Activity across Gender, *AgeG* Age across Gender, *EduG* Education across Gender. In the parentheses, the first score is for females

According to the results of univariate analysis of covariances (ANCOVA), the female participants scored insignificantly higher than the males on their total MAIA-2-N scores [*F* (1, 300) = 2.06, *p* = 0.15]. In addition, a multivariate analysis of covariance (MANCOVA) was conducted to investigate gender-based differences between males and females on the eight MAIA-2-N subscales (as dependent variables), with gender used as an independent variable in the analysis. The Box’s M assumption of the homogeneity of variance–covariance matrices was not violated [*F* (36, 35,290.67) = 1.16, *p* = 0.24]. Gender showed a significant effect on Noticing, Not-Distracting, Not-Worrying, Attention Regulation, Emotional Awareness, Self-Regulation, Body Listening, and Trusting subscales of MAIA-2-N: Hotelling's Trace *F* (8, 295) = 3.77, *p* < 0.001, *η*^*2*^ = 0.09. This effect was observed univariately on the MAIA-2-N subscales; The males scored significantly higher than the females on the Not-Worrying [F (1, 300) = 4.20, *p* = 0.041, *η*^*2*^ = 0.01], but females showed higher mean scores on Trusting subscales [F (1, 300) = 12.21, *p* < 0.001, *η*^*2*^ = 0.04]. There were no significant differences across gender on the other subscales: [*p* > 0.05, ns].

### Interoceptive awareness and related measures

Table 3 presents the Spearman correlation coefficients between subscales of MAIA-2-N and COOP/WONCA. The results indicated that the MAIA-2-N and its subscales are significantly associated with some components of COOP/WONCA. The relationship between psychological state [except with Emotional Awareness, *p* > 0.05], overall health, daily activity [except with Emotional Awareness and Body Listening, p > 0.05], and social activity [except with Emotional Awareness and Body Listening, p > 0.05], and components of interoceptive awareness was positively significant (*p* < 0.01). But physical state/fitness showed significant relations (*p* < 0.01) with Not-Distracting, Not-Worrying, and Emotional Awareness. However, the results of the correlation matrix for change in health were non-significant and uniform with all components of interoceptive awareness (p > 0.05). To sum, higher IA scores were correlated with better physical state/fitness, psychological state, overall health, daily activity, and social activity on COOP/WONCA, but not for the change in health component (*p* > 0.05). The strength of relationships for significant coefficients was low to moderate or higher (0.15 to 0.55, *p* < 0.05). An investigation of correlation coefficients between IA factors and the components of COOP/WONCA showed that the strongest correlations were between Trusting and psychological state (0.56, *p* < 0.01) and overall health (0.55, *p* < 0.01). The correlations were mainly of the same size and direction for males and females, with some exceptions (see Table 3).

It is noted in Table 3 that there is a difference in Spearman correlations between subscales of interoceptive awareness and COOP/WONCA across gender. To examine whether gender plays a moderator role, moderation analysis was run, and the results revealed that gender played a significant role in the associations between physical state/fitness and Not-Distracting (*r*_male_ = 0.19, *p* = 0.17, *r*_female_ = 0.19, *p* = 0.004, z = 2.50, *p* = 0.006), and Not-Worrying (*r*_male_ = 0.43, *p* = 0.001, *r*_female_ = 0.13, *p* = 0.04, z = 2.60, *p* = 0.005). Gender also moderated the association between psychological state and Not-Worrying (*r*_male_ = 0.03, *p* = 0.84, r_female_ = 0.37, *p* < 0.001, *z* = 2.44, *p* = 0.007), Attention Regulation (*r*_male_ = 0.06, *p* = 0.68, *r*_female_ = 0.34, *p* < 0.001, *z* = 1,94, *p* = 0.026), Self-Regulation (*r*_male_ = 0.12, *p* = 0.36, *r*_female_ = 0.43, *p* < 0.001, *z* = 2.25, *p* = 0.012), and total score of Interoceptive Awareness (*r*_male_ = 0.15, *p* = 0.28, *r*_female_ = 0.47, *p* < 0.001, *z* = 2.40, *p* = 0.008) and between overall health and Emotional Awareness (*r*_male_ = 0.44, *p* = 0.001, *r*_female_ = 0.13, *p* = 0.05, z = 2.29, *p* = 0.011).

Finally, gender also moderated the association between daily activity and Trusting (*r*_male_ = 0.56, *p* < 0.001, r_female_ = 0.34, *p* < 0.001, z = 1.84, *p* = 0.033), and between social activity and Trusting (*r*_male_ = 0.65, *p* < 0.001, *r*_female_ = 0.34, < 0.001, z = 1.86, *p* = 0.032).

According to the results, correlation coefficients between Interoceptive Awareness and age groups and education level, lower levels of Not-Distracting (*r* = -0.11, *p* < 0.05) and higher levels of Self-Regulation (*r* = 0.21, *p* < 0.01) were associated with increased age, and higher levels of Self-Regulation (*r* = 0.12, *p* < 0.05), Body Listening (*r* = 0.12, *p* < 0.05), and Trusting (*r* = 0.12, *p* < 0.05) were correlated with increased educational level (see Table 3).

As depicted in Table 3, results showed that gender played a significant moderating role in the association between age and Trusting (*r*_male_ = -0.21, *p* = 0.12, r_female_ = 0.13, *p* = 0.037, *z* = 2.30, *p* = 0.011) and between educational level and Self-Regulation (*r*_male_ = -0.11, *p* = 0.42, *r*_female_ = 0.17, *p* = 0.007, *z* = 1,87, *p* = 0.031), and educational level and Trusting (*r*_male_ = -0.20, *p* = 0.13, *r*_female_ = 0.18, *p* = 0.005, *z* = 2.55, *p* = 0.005).

## Discussion

Interoception is related to mental and physical health and is a central concept in newer theories of psychopathology [6] that could inform the future treatment of mental disorders [69]. A burgeoning line of research is revealing its associations with different transdiagnostic factors (e.g., emotion and regulation; [70, 71]) and as a possible risk factor for psychopathology [20, 21] including depression and anxiety [11, 72, 73]. The current study is an initial validation of a Norwegian translation of the MAIA-2, investigating the factor structure of the Norwegian translation, along with an exploration of the interoceptive factors’ relationship with physical state/fitness, psychological state, change in health, daily and social activities, and overall health. The study also investigated possible gender-, age-, and education-related differences in interoception and in the relationships between interoception, physical state/fitness, psychological state, change in health, daily and social activities, and overall health. The results indicate that MAIA-2-N follows the same 8-factor structure as reported for the original MAIA and MAIA-2 [31, 36]. A 6-factor structure, as reported in a Turkish translation of MAIA-2 [38], was not supported in the current study. The results indicate that the eight factors in MAIA-2-N show satisfactory internal consistency. The Cronbach's alpha coefficients were all above 0.70, and the item-total correlations were within a range of 0.44 to 0.90, exceeding the minimum acceptable value of 0.30 [74]. In model 1 (M1), the 37 items were loaded on a general, common factor of general IA to investigate the unidimensional model of a presumed latent variable and contained only random sampling errors and indicator-specific variance [75]. It was observed that the general factor model did not fit the data adequately, implying that the assumption of multidimensionality was held for the measure. This finding contradicts the prior finding of Ferentzi [76], who reported a general factor for IA in their attempt to adapt the Hungarian version of MAIA. Nonetheless, the idea of the instrument’s homogeneity has been deemed unnecessary and insufficient for understanding the practicality and theoretical usefulness of an instrument [75]. Lucke [77] asserts that the relevance of a measure is determined not by a methodological mandate of homogeneity but by the test's capacity to grasp all pertinent aspects of the entity being measured. Furthermore, our results reveal that the bifactor orthogonal model provides an adequate fit to address this complexity. The bifactor framework enables researchers to keep the concept of a single common construct (i.e., general IA) while simultaneously acknowledging multidimensionality within a construct [78]. In this way, a bifactor approach might assist clinicians in determining which symptom in an assessment may hold specific and/or general explanatory power. Finally, the fit indices of the 8-factor first-order model and the bi-factor model were compared using the principle of parsimony [79], and accordingly, the eight-factor model was selected as optimal/parsimonious.

An investigation of the correlation coefficients between the eight IA scales showed that most scales were positively related to the other scales showing weak to strong associations, indicating that the factors capture different but related interoceptive phenomena [31]. The strongest associations were between Self-Regulation (the ability to regulate distress by attention to body sensations) and Body Listening (the tendency to actively listen to the body for insight) and between Attention Regulation (the ability to sustain and control attention to body sensations) and Body Listening.

The total MAIA-2-N score was strongly related to overall health and psychological state and moderately related to physical state/fitness as assessed by the COOP/WONCA Functional chart (See Table 3). Overall, this suggests that the MAIA-2-N reflects perceptions of psychological fluctuations and patterns, along with shifts in bodily sensations, so that weaker IA is related to less favorable physical and psychological outcomes. In terms of subscale correlations, the strongest relationships were found between Trusting (the experience of one's body as safe and trustworthy) and psychological state and between Trusting and overall health. Both relationships were strongly positive, meaning that a better psychological state and overall health were related to an experience of the body as more trustworthy (and vice versa). The second strongest relationship was between Self-Regulation and better psychological state (lower distress) and overall health. However, Self-Regulation was only significantly related to the psychological state of females. Furthermore, Self-Regulation increased with age and education. Finally, individuals with greater levels of education showed higher levels of Body Listening and Trusting.

Interoception is implicated in both psychological and somatic disorders, with a gender gap; nevertheless, sex differences are frequently overlooked [27]. Some preliminary studies indicate gender differences in interoception [27, 40, 80] which may also be a contributing factor to the increased risk of psychopathology among females [26]. Investigations of gender differences in interoception in our sample indicated that females scored higher on Trusting than males indicating that they felt more at home in their bodies than the male participants. This finding is inconsistent with the previous research indicating higher rates of bodily trust among males [27]. Furthermore, the results of our study showed that males reported higher scores on the Not-Worrying (the tendency not to worry or experience emotional distress related to sensations of pain or discomfort) factor, indicating a higher inclination to self-reported low worry about feelings of pain or discomfort compared to the females in the sample. This result replicates previous findings that males scored higher on not-worrying than females [27]. A pattern of greater interoceptive attention and higher worry about interoceptive signals could represent an increased risk profile for negative psychological consequences in females compared to males, particularly when considering the previously reported lower interoceptive accuracy in females [26, 28]. Whether such differences reflect on gender-stereotypical response patterns that stem from social influences or represent physiological or perceptual gender differences [26, 81, 82] is still unclear. Not unexpectedly, our results indicated further gender differences when looking at the moderating role of gender on the relationship between IA components and COOP/WONCA subscales. Specifically, the results indicate a significant moderating role of gender in the association between Not-Worrying and physical state/fitness for both males and females. This association was positive and stronger for males meaning that males who estimated their physical state/fitness as more positive also reported a tendency to worry less about pain or discomfort. This observation is perhaps congruent with the pain perception literature, wherein researchers frequently documented greater pain ratings by females than males across diagnoses [83]. In contrast, another moderating role for gender was found between Not-Worrying and psychological state for females but not for males, meaning females who reported a better psychological state also indicated less interoceptive worrying. While these results should be replicated in larger samples, they suggest that a state of lower interoceptive worry is more strongly connected to positive physical state/fitness for males and a positive psychological state for females. Gender also played a moderating role in the relationship between physical state/fitness and Not-Distracting; however, the observed association was only significant and positive for females. Additionally, further investigation of the moderating effects of gender revealed a significantly strong positive relationship between the factors of Self-Regulation and psychological state for females but not for males. Previous research in this area has been scarce and mixed. Also, this finding aligns with the work of Millon & Shors [84], who found that females who reported a higher number of dysfunctional symptoms related to mental health had a lower capacity to recognize and trust their physical sensations, as well as calibrating emotions and thoughts related to these sensations. On the other hand, Fazekas et al. [85] found no variance in factors assessing IA and self-regulation among female and male respondents. The moderating effects of gender also demonstrated a significantly strong positive relationship between the factors of Attention Regulation and psychological state for females but not for males. Also, the association between MAIA-2-N total score and psychological state was significantly moderated by gender, and this effect was strong, positive, and significant for females but insignificant for males. Gender moderated the positive association between daily activity and trusting for both males and females, and this association was stronger for males. Finally, gender moderated the association between social activity and Trusting, and this link was positive and significant for both genders, although it was stronger for males.

We inspected the moderating role of gender between IA subscales and education as well as age. Gender moderated the link between Self-Regulation and education. This association was significant for females in the sense that females with higher educational status reported higher interoceptive self-regulation. Our findings also showed that gender moderated the associations between bodily trust (Trusting) and age and between bodily trust and education. This association was significant for the female group, and the direction of the associations suggests that as females age, their bodily trust increases. Similarly, the results showed that levels of bodily trust also increased with higher education in females.

Despite the scarcity of research on the link between psychopathology and bodily trust, the ability to trust the body has been noticed and accentuated in the literature on suicide and life-threatening behavior. Gioia et al. [86] found that suicidal ideation and non-suicidal self-injury were both predicted by decreased bodily trust, which suggests a possible common contributing risk factor. Also, Rogers et al. [87] demonstrated that those with a record of suicidal behavior (ideation, attempt, etc.) had significantly lower trust in their bodily sensations than those with no history of suicidal behavior. Finally, Duffy et al. [88] demonstrated that bodily trust serves as a moderator between exercise dependence and suicidal behavior and that for at-risk individuals, feelings of not trusting one's own body might increase suicide risk. The findings in this line of research are promising and enlightening, and future research may explore the panorama of IA, including bodily trust, and how it can increase or reduce the risk for suicidal behaviors further.

### Limitations and future directions

Even though this research has several strengths, it has a number of limitations that should be addressed. First, the sample was drawn from a well-educated population, which may limit the overall generalizability of the results. However, we assume that our sample represents a diverse and broad Norwegian population, particularly considering the generally high level of education in Norway, with 36% of people over 16 years of age having completed higher education [89]. Second, the present research relies on a self-report measure (MAIA-2-N) to assess participants' IA. Although participants experience of IA is valuable in itself, it is unclear to what extent self-reported IA conforms to IA as assessed by objective methods. Future studies should focus on this matter, taking into account the construct's multidimensionality [90]. Thus, in order to investigate IA, numerous concrete tests (including, for example, physiological, behavioral, and neural data) that may correspond to the various elements of IA as measured by the MAIA-2-N could be used. It is a further limitation that the gender balance was uneven, with few males (19%) represented. Of note, we also attempted gender invariance analyses, but due to sample constraints, we were unable to generate appropriate results to report and interpret these. The results must be considered an initial validation of MAIA-2 in Norwegian, and the results require further replication in more extensive and diverse samples. Since the most robust associations were identified between Trusting and overall health and psychological state it appears that particularly the concept of bodily trust requires more focus and in-depth consideration in future studies.

## Conclusion

The current study demonstrated that the MAIA-2-N, the Norwegian version of the MAIA-2, shows appropriate psychometric characteristics. Our findings confirmed an 8-factor structure similar to the parent model proposed in the original studies [31, 36]. Given that the MAIA and, consequently, MAIA-2 design presupposed a multidimensional nature of IA, this study provides additional evidence that the measure incorporates heterogeneity and corroborates the existence of a multidimensional nomological network of IA concepts, as seen in the 8-factor-structure. In addition, the MAIA subscales of Not-Distracting and Not-Worrying, which had previously been questioned as lacking sufficient reliability and having a problematic factor structure, were amended favorably in MAIA-2 [36]. This was further corroborated in our MAIA-2-N adaptation, where their reliability was found to be appropriate. To recapitulate, the current study demonstrated that the MAIA-2-N is an adequate measure of IA in the Norwegian population and that it can be implemented in research and clinical contexts. It also showed interesting relationships between aspects of IA and physical state/fitness and psychological state and health, including effects of gender that warrant further exploration.

## Data Availability

The datasets generated and/or analyzed during the current study are not publicly available before the end of the project in December 2024, but are available from the corresponding author on reasonable request. After the conclusion of the project the data will be made available through a restricted data repository from the Norwegian government (Norwegian Centre for Research Data). The MAIA-2-N is available through the MAIA official website, https://osher.ucsf.edu/research/maia.

## Footnotes

1 We have referred to this scale as "psychological state" throughout this article, and our readings of its contents support that.
